# Analysis and reporting of individually randomised controlled trials with clustering in one arm only: how should we do it?

**DOI:** 10.1186/1745-6215-16-S2-P139

**Published:** 2015-11-16

**Authors:** Stephen Walters, Ellen Lee, Laura Flight, Laura Mandefield, Munya Dimario, Annabel Allison

**Affiliations:** 1 University of Sheffield, Sheffield, UK

## 

In an individually randomised controlled trial (iRCT) where the intervention is delivered by a health professional/therapist it seems likely that the effectiveness of the intervention could depend on the skill of the therapist delivering it. This leads to a potential clustering of the outcomes for the patients treated by the same therapist.

If outcomes are clustered then the usual statistical methods for analysing RCT data may not be appropriate as they assume that outcomes observed on different participants are independent.

In some iRCTs there is clustering in only one arm; where the therapist only treats participants in one arm of the trial but there is no equivalent clustering in the control arm.

Several strategies have been proposed for this problem: ignoring the clustering; imposing clusters in the control arm and fitting a fixed or random effects model, or using a partially clustered approach where only the clustering in the treatment arm is modelled.

This talk will describe and compare the statistical methods for analysing continuous outcomes from an iRCT with some element of clustering in one arm. Four confirmatory case studies (with a combined 1,167 randomised participants) will be analysed using each of the methods: specialist clinics for the treatment of venous leg ulcers (Morrell et al. 1998), acupuncture for low back pain (Thomas et al. 2006), cost-effectiveness of community postnatal support workers (CPSW) (Morrell et al. 2000), and Putting Life in Years (PLINY) (Mountain et al. 2014). Recommendations for the best and most practical approaches will be made.

